# A case of non-occlusive mesenteric ischaemia caused by pelvic fracture due to fall trauma

**DOI:** 10.1186/s40792-020-01046-x

**Published:** 2020-12-09

**Authors:** Yuichiro Furutani, Kaname Ishiguro, Masato Tokuraku, Hitoshi Moritomo

**Affiliations:** Department of Surgery, Noto General Hospital, Surgery, Abe 6-4, Fujihashi, Nanao, Ishikawa 910-8526 Japan

**Keywords:** Non-occlusive mesenteric ischaemia (NOMI), Pelvic fracture, Traumatic bleeding

## Abstract

**Background:**

Non-occlusive mesenteric ischaemia (NOMI) is a condition in which intestinal ischaemia arises due to spasms of peripheral blood vessels; however, there is no obstruction of the main arteries. Risk factors include hypertension, diabetes, and increasing age, but the traumatic injury triggering NOMI onset is rarely reported. We report a case of NOMI caused by a pelvic fracture due to a fall injury.

**Case presentation:**

A 77-year-old man was transported to the hospital due to a fall injury. CT revealed a pelvic fracture and a haematoma in the pelvic extraperitoneal space. The next day, the patient developed shock, and CT revealed an increase in haematoma size. Both internal iliac arteries were embolized by transcatheter arterial embolization (TAE). The next day’s CT revealed intestinal necrosis of the ascending colon, and emergency surgery was planned. During surgery, necrosis was identified in the serosa of the ascending, transverse, and sigmoid colon. We performed subtotal excision from the ascending colon to the sigmoid colon. On postoperative day 10, melena was observed, and CT revealed partial thickening of the small intestine and a decrease in the contrast effect. Considering the post-total colectomy and general condition, we proceeded with conservative treatment. Over time, the patient developed liver and renal dysfunction and died 16 days after surgery.

**Conclusions:**

We experienced a case of NOMI caused by bleeding from a pelvic fracture. It is important to keep in mind the risk of developing NOMI in traumatic bleeding to avoid missing this diagnosis.

## Background

Non-occlusive mesenteric ischaemia (NOMI) is a condition in which intestinal ischaemia arises due to spasms of peripheral blood vessels; however, there is no obstruction of the main arteries. Risk factors include hypertension, diabetes, and increasing age, but traumatic injury triggering its onset has rarely been reported. Pelvic fractures generally cause extensive blood loss, even in trauma, and vital signs are likely to become unstable. Herein, we report a case of NOMI caused by a pelvic fracture due to a fall injury.

## Case presentation

A 77-year-old man fell from a height of approximately 3 m into the gutter and struck his pelvis. He was found immobile and was rushed to our hospital.

Medical history: none.

Physical findings: Consciousness was clear, blood pressure was 100/70 mmHg, heart rate was 127/min, SpO2 was 97%, and no injury was noted on either the head or abdomen.

As shown in Table 1, blood work (day 1) showed Hb12.7 g/dl, but no increase in cell ectopic enzymes was observed.

**Table 1:** Blood chemistry

	Day 1	Day 2	Day 3	Unit
WBC	14,770	25,040	21,530	/μl
RBC	458	367	490	/μl
Hb	12.7	10.3	14.3	g/dl
Ht	40.6	33.5	40.3	%
Plt	14.8 × 10^4^	12.9 × 10^4^	8.1 × 10^4^	/μl
AST	32	116	2363	IU/l
ALT	13	70	2233	IU/l
LDH	282	432	3181	IU/l
ALP	208	194	189	IU/l
CPK	743	1895	4122	IU/l
TB	0.8	–	3.0	mg/dl
BUN	16	20	30	mg/dl
Cre	0.88	1.69	1.57	mg/dl
Alb	3.7	3.5	4.4	g/dl
Na	140	145	140	mEq/l
K	3.7	3.5	4.4	mEq/l
Cl	106	105	105	mEq/l
CRP	0.12	–	8.44	mg/dl

*WBC* white blood cell, *RBC* red blood cell, *Hb* haemoglobin, *Ht* haematocrit, *Plt* platelet, *AST* aspartate aminotransferase, *ALT* alanine aminotransferase, *LDH* lactate dehydrogenase, *ALP* alkaline phosphatase, *CPK* creatine phosphokinase, *TB* total bilirubin, *BUN* blood urea nitrogen, *Cre* creatinine, *Alb* albumin, *CRP* C-reactive protein

**Fig. 1:**
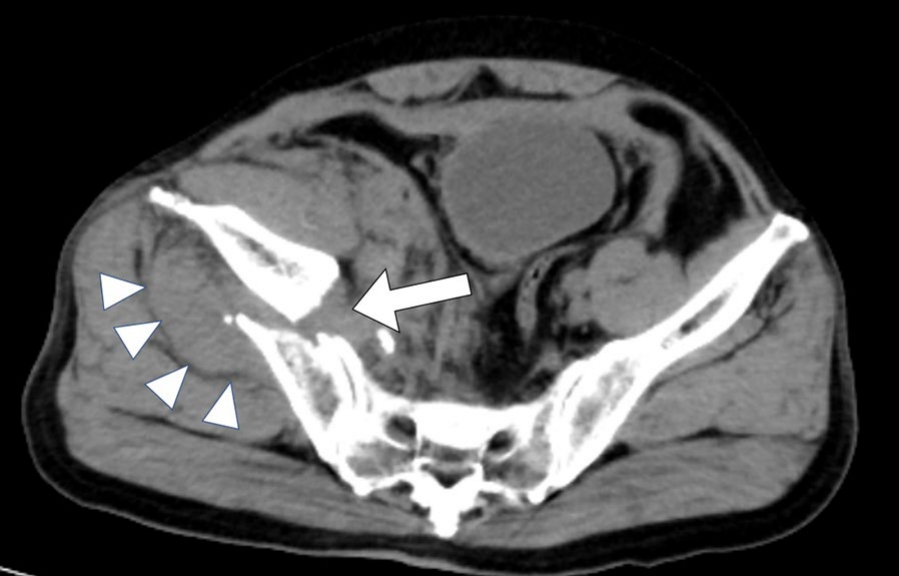
CT findings on day 1. CT revealed a right pelvic fracture (arrow) and a haematoma (arrowhead) in the pelvic extraperitoneal space

CT findings on day 1 revealed a right pelvic fracture and a haematoma in the pelvic extraperitoneal space (Fig. 1). Neither free air nor ascites was observed in the abdominal cavity, and no damage was found to the intestinal tract. CT findings on day 2 showed the haematoma in the pelvic extraperitoneal space had increased in size from the previous day, and active bleeding was suspected. Blood work from day 2 demonstrated progression of Hb10.3 and anaemia, even after transfusion of 4 units of red blood cells (Table 1). For vitals, blood pressure was 78/37 mmHg, and heart rate was 144 bpm, indicating shock, and active bleeding due to the pelvic fracture was suspected. Therefore, transcatheter arterial embolization (TAE) was adopted. Blood flow in the pelvis is supplied from both sides, and the presence of traffic branches may require TAE of the internal iliac arteries on both sides depending on the situation. Angiography identified the responsible vessel, which had embolized the bilateral internal iliac arteries, and confirmed haemostasis.

**Fig. 2:**
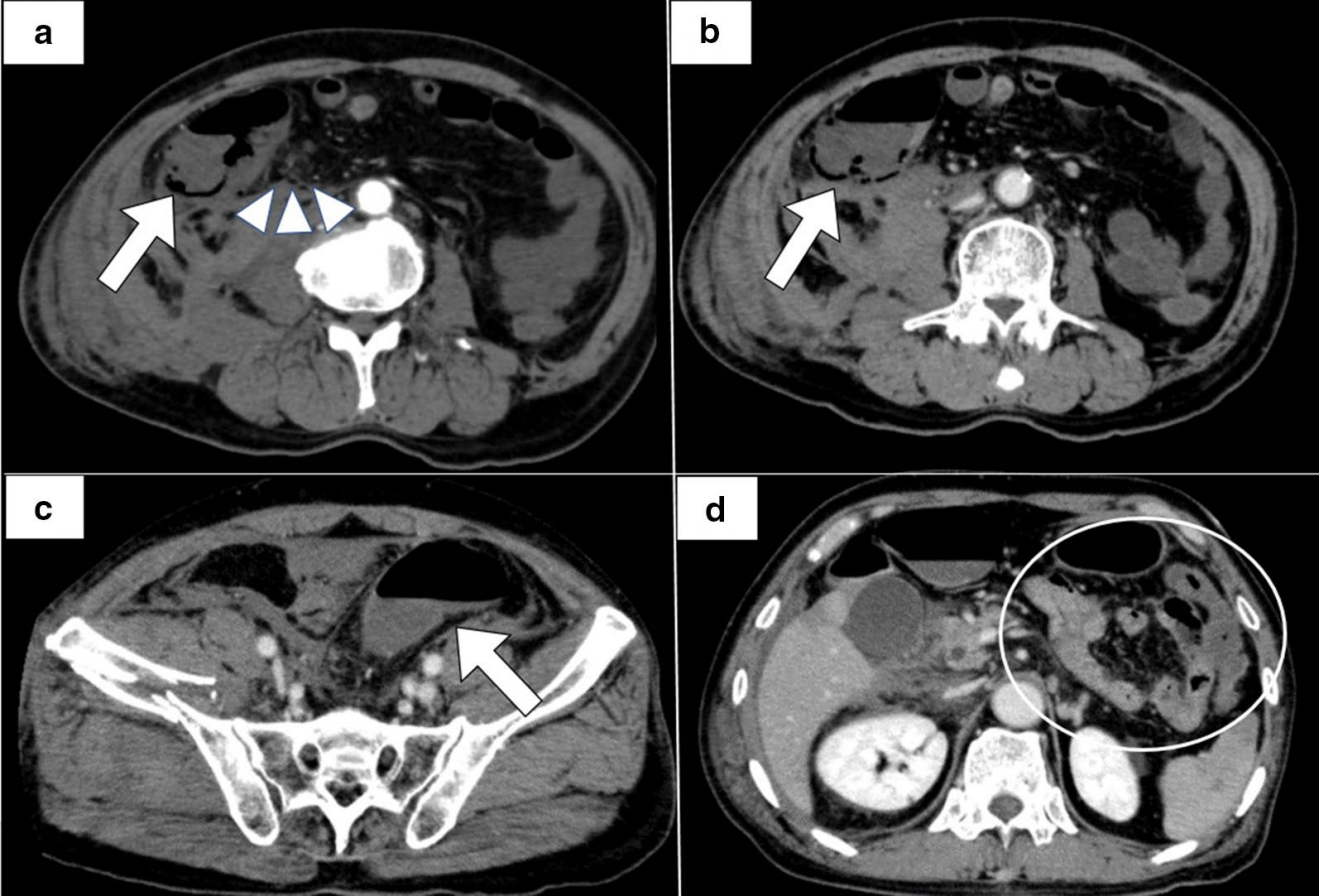
CT findings on day 3: **a** the early arterial phase; **b**–**d** the portal venous phase. **a** Thickening ascending colon wall, intramural emphysema (arrow), and a slight amount of air in the mesenteric vein (arrowhead). **b** Intramural emphysema in ascending colon (arrow). **c** Dilated sigmoid colon (arrow). **d** Small intestine with no evidence of necrosis (circle)

CT findings on day 3 revealed thickening of the ascending colon wall, intramural emphysema, and a slight amount of air in the mesenteric vein. In addition, a small amount of ascites was observed near the ascending colon. There was no problem with the contrast effect of the small intestine, and the sigmoid colon was dilated, but there were no obvious necrotic findings. (Fig. 2).

Blood work on day 3 revealed elevated levels of cytopathic enzymes: AST 2363 IU/l, ALT 2233 IU/l, LDH 3181 IU/l, and CPK 4122 IU/l. Based these findings, we rendered a diagnosis of necrosis of the ascending colon and performed emergency surgery.

Intraoperatively, necrosis was observed in the serosa of the ascending, transverse, and sigmoid colon. Subtotal resection was performed from the ileocecal region to the sigmoid colon. After confirming that there is no necrosis in the mucosa of cut end of small intestine, ileostomy was constructed. Although a slight ischaemic change such as edema and mild redness was observed in the serosa of the entire small intestine, we decided to preserve the entire intestine.

**Fig. 3:**
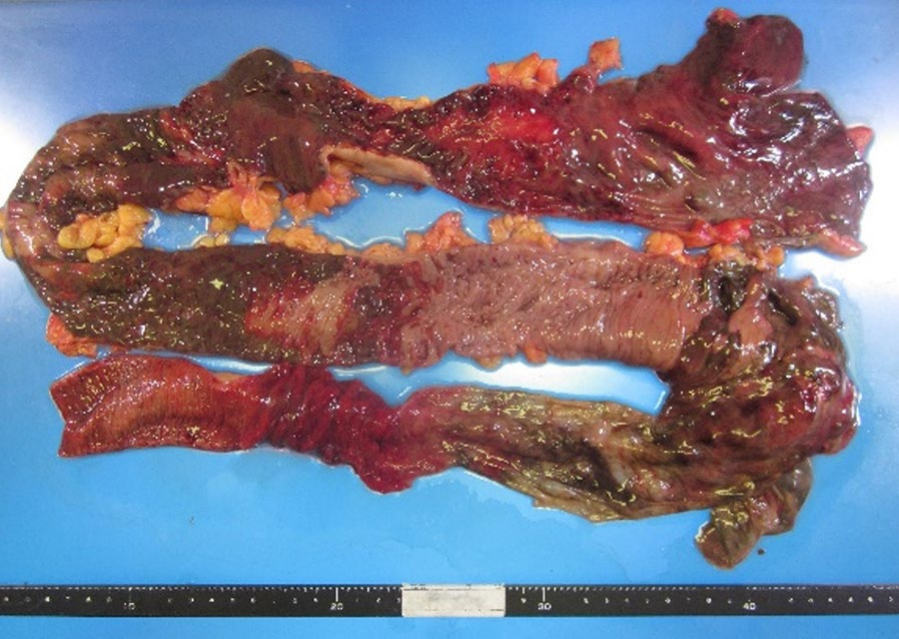
Excised specimen findings. The excised specimen showed scattered necrosis in the ascending, transverse, and sigmoid colon

Excised specimen findings showed scattered necrosis in the ascending, transverse, and sigmoid colon (Fig. 3).

Pathological findings indicated necrosis throughout all layers of the intestinal wall in the ascending, transverse, and sigmoid colon.

**Fig. 4:**
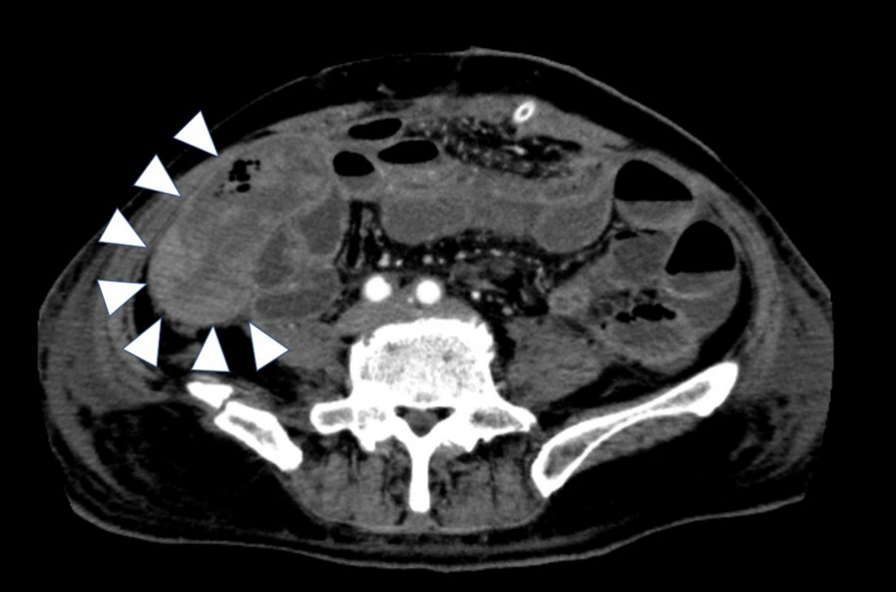
CT findings 10 days after surgery. CT showed wall thickening and a decrease in contrast effect in some small intestines (arrowhead)

Postoperatively, the patient began drinking water 2 days after surgery. However, 6 days after surgery, CT revealed a dilated small intestine, which we diagnosed as paralytic ileus. Subsequently, the paralytic ileus did not improve, and melena was observed 10 days after surgery. CT performed 10 days after surgery revealed extensive small bowel dilation and niveau. In addition, wall thickening and a decrease in contrast effect were observed in partial small intestines (Fig. 4).

We diagnosed haemorrhage due to necrosis of the residual small intestine. However, due to the postoperative total colectomy and deterioration of general condition, it was decided that intestinal resection was impossible, and treatment with blood transfusion and haemostatic agents was planned. At that time, the colour of stoma did not change greatly and there were no necrosis sites.

The patient's condition gradually worsened even after treatment, and over time, he developed liver and renal dysfunction and died 16 days after surgery.

### Discussion

NOMI is a disorder that causes irreversible ischaemia in the intestine, resulting in intestinal necrosis, despite the absence of organic obstruction in the mesenteric artery trunk. This phenomenon was first reported by Heer [1] and includes the following characteristics: (1) no obstruction in the mesenteric arteries or veins governed by intestinal necrosis, (2) segmental discontinuity of intestinal ischaemia and necrosis, and (3) pathological evidence of intestinal haemorrhage and necrosis. We define NOMI as satisfying these conditions. From 12 to 25% of acute intestinal ischaemia is thought to be due to NOMI in Europe and America [2, 3]. Various triggers increase hypoxia of the intestinal tract tissue, reduce cardiac output, and decrease circulating blood volume. As a result, the sympathetic nerves in the peripheral blood vessels of the mesenteric artery overreact, causing vasospasms and intestinal ischaemia. Since these spasms occur at random, the ischaemic area is sporadic. Fogaty [4] suggests that severe congestive heart failure, digitalis intoxication, and blood concentration are important factors affecting this disorder and that dehydration is significantly related to vasopressin due to low cardiac output, haemorrhage, and shock. He asserts that vasopressin and angiotensin increase in the blood due to low cardiac output, haemorrhage, shock, etc., causing catecholamine-induced spasms of the mesenteric artery and resulting in NOMI. In addition, the general risk factors for NOMI are increased age, heart disease, arrhythmia, cerebrovascular disease, diabetes, burns, dialysis, dehydration, haemorrhage, and pancreatitis [5–9]. In any case, the basis of the pathology is considered a decrease in circulating blood volume. Pelvic fractures generally cause excessive bleeding in trauma, anywhere from 1000 to 4000 ml [10]. In our patient, bleeding from a pelvic fracture caused a rapid decrease in circulating blood volume, and at one point, the patient was in shock. TAE was performed for active bleeding, and NOMI developed the next day, although haemostasis was attained. When a pelvic fracture with a large volume of blood loss occurs, the risk of NOMI onset should not be ignored.

NOMI has no specific symptoms. Some of the many non-specific symptoms include abdominal pain, vomiting, abdominal distension, and melena, but these symptoms are often mild during onset. In particular, it is difficult to identify these symptoms in cases of sedation, analgesia, and consciousness disorder. In these cases, diagnosis is likely to be delayed. Similarly, in our case, it was difficult to diagnose intestinal ischaemia based on clinical symptoms, because analgesics are used for pelvic fractures, and systemic contusions are caused by trauma. Blood work showed an increase in deviant enzymes, but the diagnosis was made even more difficult after TAE was performed for active bleeding. In our case, follow-up CT was performed to identify active bleeding, but it is important to consider CT examination as necessary, keeping in mind that NOMI is caused by bleeding from trauma.

When NOMI is considered as a diagnosis, one of the treatments is injection of a vasodilator into the responsible blood vessel using angiography [11], but this method is limited by equipment availability and the specific situation. In our case, a vasodilator could not be used due to instability of vital signs from bleeding. If intestinal necrosis is already suspected, as in our case, immediate surgical intervention is needed. At the time of surgery, it is important not only to remove the necrotic intestine but also to evaluate the viability of the remaining intestine. Intestinal ischaemia can be extensive postoperatively, and care must be taken in determining the extent of resection. When deciding the extent of resection, there is a method of observing changes in the colour tone of the mucous membrane using an intraoperative lower gastrointestinal endoscope and blood flow evaluation by fluorescein fluorescence [12, 13]. However, these methods may be difficult depending on equipment availability and the situation. In our case, we found clear discontinuous necrosis in the serosa of the caecum, transverse colon, and sigmoid colon. Therefore, we decided to remove the intestinal tract from the ascending colon to the sigmoid colon. Since only diffuse and mild oedema and redness in the small intestine were noted and no obvious necrosis was observed, we decided to preserve it and constructed an artificial anus in the terminal ileum. However, the small intestines became necrotic after a few days, eventually resulting in widespread intestinal necrosis. Of note, the prognosis cannot be judged only by the surgical findings. If long-term dilatation of the small intestine is observed after heavy bleeding, it is important to consider intestinal necrosis rather than assuming it as paralytic ileus. In that case, it is necessary to comprehensively judge clinical symptoms, vital signs, and imaging findings, but the diagnosis is very difficult. Importantly, pelvic fractures are typically accompanied by high blood loss, so even if active bleeding subsides and vital stability is obtained, NOMI may develop over time.

## Conclusion

We treated a case of NOMI caused by bleeding from a pelvic fracture. It is important to bear in mind the risk of developing NOMI in traumatic bleeding so as not to miss this diagnosis.
